# I Am So Tired… How Fatigue May Exacerbate Stress Reactions to Psychological Contract Breach

**DOI:** 10.3389/fpsyg.2018.00231

**Published:** 2018-03-06

**Authors:** Safâa Achnak, Yannick Griep, Tim Vantilborgh

**Affiliations:** ^1^Work and Organizational Psychology, Vrije Universiteit Brussel, Brussels, Belgium; ^2^Department of Psychology, University of Calgary, Calgary, AB, Canada; ^3^Division of Epidemiology, Stress Research Institute, Stockholm University, Stockholm, Sweden

**Keywords:** psychological contract breach, stress, negative emotions, fatigue, moderated mediation

## Abstract

Previous research showed that perceptions of psychological contract (PC) breach have undesirable individual and organizational consequences. Surprisingly, the PC literature has paid little to no attention to the relationship between PC breach perceptions and stress. A better understanding of how PC breach may elicit stress seems crucial, given that stress plays a key role in employees' physical and mental well-being. Based on Conservation of Resources Theory, we suggest that PC breach perceptions represent a perceived loss of valued resources, subsequently leading employees to experience higher stress levels resulting from emerging negative emotions. Moreover, we suggest that this mediated relationship is moderated by initial levels of fatigue, due to fatigue lowering the personal resources necessary to cope with breach events. To tests our hypotheses, we analyzed the multilevel data we obtained from two experience sampling designs (Study 1: 51 Belgian employees; Study 2: 53 US employees). Note that the unit of analysis is “observations” rather than “respondents,” resulting in an effective sample size of 730 (Study 1) and 374 (Study 2) observations. In both studies, we found evidence for the mediating role of negative emotions in the PC breach—stress relationship. In the second study, we also found evidence for the moderating role of fatigue in the mediated PC breach—stress relationship. Implications for research and practice are discussed.

## Introduction

The psychological contract (PC) forms the foundation of the employee—employer relationship because it encompasses beliefs about reciprocal obligations between employees and their employer based on explicit and implicit promises (Rousseau, 2001). An arguably more interesting component of the PC pertains to the fact that employees often perceive that their organization failed to fulfill one or more obligations, leading them to experience a PC breach and negative emotions following this PC breach (Morrison and Robinson, 1997). Research has shown that the prevalence of PC breach is quite high, with employees perceiving PC breach at least once a week (Conway and Briner, 2002). Although the relationship between PC breach and employee attitudes or behaviors has been documented extensively (for a meta-analyses see Zhao et al., 2007), far fewer studies have explored its influence on employee well-being in terms of stress.

The lack of attention for stress in the PC literature is surprising both from a theoretical and a practical point of view. Based on Conservation of Resources Theory (COR; Hobfoll, 1989), stress can be considered a plausible outcome of the negative emotions resulting from PC breach because it represents a loss or potential loss of valued resources, which is considered a stressful event (Restubog et al., 2013). From a practical standpoint, stress can have detrimental consequences both for the individual and the organization. For instance, (chronic) experiences of stress are associated with, inter alia, poor mental health (Godin et al., 2005), hypertension (Markovitz et al., 2004), depression, chronic fatigue syndrome (Marin et al., 2011), burnout (Maslach et al., 2001), as well as absenteeism (Undén, 1996; Guglielmi and Tatrow, 1998). These detrimental well-being and health outcomes subsequently negatively impact the organization through, for example, increased sick leave and turnover, and reduced performance. A better understanding of the relationship between PC breach, negative emotions and stress is therefore imperative to avoid the detrimental consequences resulting from stress.

**Figure 1:**
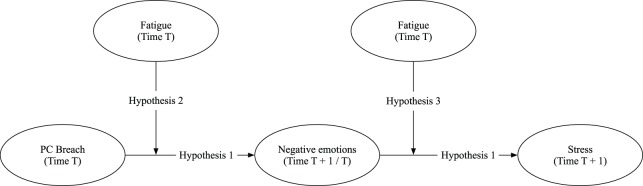
Proposed moderated mediation model.

So far, only a limited number of scholars has demonstrated a positive relationship between PC breach and well-being indicators such as emotional exhaustion (Johnson and O'Leary-Kelly, 2003; Cantisano et al., 2007), anxiety and depression (Conway and Briner, 2002; Slack, 2004), and burnout (Brown, 2007). One such noteworthy study is that of Gakovic and Tetrick (2003), which—to date—is the only study that examined the relationship between PC breach and strain resulting from perceived stress. Our goal is to extend this important study, while taking into account some of its limitations. First, Gakovic and Tetrick's research (Gakovic and Tetrick, 2003) did not include the influence of individual factors. However, research has shown that stress processes are not invariant and mainly influenced by personal factors such as coping (Folkman, 2013), personal resources (Hobfoll, 1989), and negative affect (Clark and Watson, 1988). The relevance of including individual factors in examining stress reactions has been recognized by several researchers (e.g., Parkes, 1994; Jepson and Forrest, 2006). Therefore, we propose that the relationship between PC breach and stress is mediated by evoked negative emotions, which in turn is moderated by employees' initial levels of fatigue (see Figure 1). By examining this moderated mediation model, we offer a more complete and accurate understanding of the dynamic relationship between these concepts. Second, Gakovic and Tetrick (2003) focused on emotional exhaustion and job dissatisfaction, which represent the experience of strain *resulting* from stress, rather than the experience of stress itself. Therefore, we will focus on general work stress which is considered to be distinct from job dissatisfaction (Stanton et al., 2001) and influenced by different factors (Fairbrother and Warn, 2003). Moreover, following COR Theory, we propose that an actual or a potential resource loss, due to PC breach, will trigger stress as a consequence of the experienced negative emotions following PC breach. Only when these negative emotions are left unattended over time, will they lead to emotional exhaustion and potentially other maladaptive consequences (Hobfoll, 1989).

Building on Gakovic and Tetrick's (2003) call for a further integration of the PC concept in the stress literature, the current 2-study paper investigates the dynamics that relate perceptions of PC breach to negative emotions and experiences of stress. By doing so, we are contributing to the literature in two important ways. First, we focus on within-person processes as opposed to between-person differences. Despite calls for more attention to within-person processes in PC research (Conway and Briner, 2009), the majority of the literature operates under the sole assumption that employees who perceive a high level of PC breach are characterized by higher levels of negative emotions and stress than people who perceive a low level of PC breach (i.e., between-person differences). However, by adopting a within-person process perspective we are able to examine the arguably far more important question as to *how* perceiving PC breach elicits *increases* in stress resulting from negative emotions within the same individual, compared to when that individual does not perceive PC breach. Moreover, it allows us to test time-lagged relationships and establish the direction of the PC breach—negative emotions—stress relationship. Second, simultaneously examining mediating (negative emotions) and moderating (fatigue) mechanisms allows a more detailed understanding of the interplay between PC breach and stress. Therefore, our second goal is to extend previous research by integrating the PC concept in the stress literature.

## Theoretical framework and hypotheses

### Psychological contract breach, negative emotions, and stress

Organizations are not always able, or willing, to fulfill all of their obligations toward employees, resulting in PC breach perceptions (Morrison and Robinson, 1997). In addition, employees may believe that their employer is breaching certain obligations, even in the absence of actual PC breaches. When one or more organizational obligation(s) are breached, an emotional and affective reaction might be evoked. This reaction is most commonly explained by Affective Events Theory (AET; Weiss and Cropanzano, 1996). According to AET, PC breaches are affective events that elicit a strong emotional reaction. These emotional reactions are termed feelings of violation and entail a mix of negative emotions such as betrayal, resentment, anger, and disappointment (Morrison and Robinson, 1997). These negative feelings may in turn trigger unfavorable attitudes and behaviors such as reduced performance and trust in the organization, and increased job dissatisfaction and turnover intentions (for a meta-analyses see Zhao et al., 2007). However, according to Morrison and Robinson (1997), not every PC breach will elicit feelings of violation. For example, if an employee perceives a PC breach but believes that this breach has few implications for him/her, (s)he may experience a limited emotional reaction, or no emotional reaction at all. Morrison and Robinson (1997) explain this process by arguing that employees who perceive a PC breach will go through a cognitive appraisal process through which they evaluate the resources they may have lost as a consequence of this PC breach. Indeed, according to COR theory (Hobfoll, 1989), employees have a need and desire to maintain valuable resources. Resources are “*those objects, personal characteristics, conditions, or energies that are valued by the individual or that serve as means for attainment of these objects, personal characteristics, conditions, or energies”* (Hobfoll, 1989, p. 516).

Stress occurs in any circumstance involving loss, potential loss, or failure to gain resources (e.g., time, money, health, relationships; Hobfoll, 1989; Kiazad et al., 2014). These stress reactions are characterized by physiological (e.g., increases in blood pressure and heart rate), psychological (e.g., tension, anxiety, depression, and psychological fatigue), and behavioral (e.g., absenteeism and turnover) indicators (House, 1974; Schuler, 1980). From a COR perspective, it could be argued that employees evaluate their perceptions of PC breach by assessing whether the resources threatened by the PC breach are important or not (Hobfoll, 1989). When the outcome of this evaluation point toward a threat of valued resources, employees tend to experience a strong negative emotional reaction (Zhao et al., 2007). For example, if the organization fails to provide reasonable guarantees of short-term employment (i.e., PC breach), employees' resources, and potentially their ability to preserve their current personal lifestyle, may be threatened (Restubog et al., 2013). Following COR theory (Hobfoll, 1989), the negative emotions resulting from this uncertainty will inherently lead employees to experiences higher stress levels. Put differently, the PC may be considered as obligations to exchange resources between two parties and a PC breach as a (potential) loss of these valued resources which will elicit negative emotions, and in turn, lead to stress.

Furthermore, PC breach can also be perceived when the organization over-fulfills one or more obligations toward an employee (Turnley and Feldman, 1999). However, because resources loss is disproportionately weighted compared to gain, loss tends to have a greater negative impact on emotions and stress (Hobfoll and Wells, 1998). Nevertheless, it has been argued that breaches in terms of over-fulfillment of the PC can also, albeit depending on the type of the exceeded inducement, lead to negative outcomes (Lambert et al., 2003; Vantilborgh et al., 2014). For example, inducements such as pay, recognition, and relationships will lead to positive outcomes if they are received in excess, whereas others, such as task variety, skill development, and career training, will have an unfavorable impact (Lambert et al., 2003). Therefore, we propose that exceeded inducements that are associated with negative outcomes will also lead to negative emotions, and in turn, elevated stress levels.

*Hypothesis 1: Negative emotions will mediate the relationship between PC breach and stress*.

### The moderating role of fatigue

When faced with an important loss, or threat, of valued resources, employees will actively engage in coping strategies to reduce the negative impact of loss (Hobfoll, 2002) However, actively coping with problems in stressful circumstances, requires either specific resources to respond adequately to the situation or the possession of resources that can provide access to the resources needed to adequately respond to the situation. As such, employees who possess a wide range of (energetic) resources (e.g., energy, time, money, knowledge, autonomy, etc.) will develop less negative emotions and stress in the face of PC breach because they are able to find alternative resources to (partially) offset the loss (Hobfoll, 2002). For example, an employee who has sufficient energy may decide to ask colleagues for help to offset the negative consequences of the breach and avoid stress. In contrast, when employees lack resources, they may end up in a state of fatigue, consequently preventing them from coping efficiently with an affective work event such as PC breach. Fatigue is a natural adaptive response, whereby one is shifting one's attention from the external environment toward internal signals in an attempt to conserve the remaining resources. Although fatigue has been traditionally seen as an outcome of stressful events (Motowidlo et al., 1986; Godin et al., 2005), fatigue can also be considered a moderator in the mediated PC breach—stress relationship. More specifically, according to COR Theory (Hobfoll, 1989), the levels of energy available when facing an affective work event such as a PC breach, will influence the intensity of the emotional reaction. Hence, if the initial level of fatigue is high, negative emotions following PC breach are expected to increase because the perception of PC breach consumes already depleted resources, which in turn thus contributes to a further accumulation of resource loss and threat (Hobfoll, 2001). Therefore, we suggest that an employee who is fatigued (i.e., employees with a lack of energetic resources) at the moment (s)he perceives a PC breach, will experience more intense negative emotions compared to moments when that employee was not fatigued. We thus hypothesize the following:

*Hypothesis 2: The relationship between psychological contract breach and negative emotions is moderated by fatigue, with stronger effects when fatigue is high*.

Moreover, it could also be argued that the relationship between negative emotions and stress will vary depending on the amount of available (energetic) resources. That is, when employees are confronted with negative emotions, they must engage in emotion-regulation strategies to reinterpret and regulate these negative emotions; emotion-regulation strategies that are known to be energy-consuming (John and Gross, 2004). If an employee is fatigued at the moment (s)he is confronted with these negative emotions, that employee may experience a downward spiral of energy loss, preventing him/her from using adequate resources to use adaptive coping styles that facilitate the stress resolution process (Hobfoll, 2001). In line with this, we suggest that initial fatigue levels will also moderate the relationship between the experience of negative emotions and stress in such a way that an employee who is fatigued at the moment (s)he experiences negative emotions, will experience higher levels of stress compared to moments when that employee was not fatigued. We thus hypothesize:

*Hypothesis 3: The relationship between negative emotions and stress is moderated by fatigue, with stronger effects when fatigue is high*.

## Study 1

### Method

#### Procedure

We employed an experience sampling method (ESM) in which employees from various Belgian organizations (private and public sector) reported on their momentary experiences at two moments per day for 10 consecutive working days. ESM allows to reduce the bias and error of retrospective reporting, which might lead to questionable responses due to selective memory processes (Alliger and Williams, 1993; Fisher and To, 2012). Moreover, ESM is useful to capture daily fluctuations and short-term dynamics of behaviors and attitudes, which makes it possible to study within-person processes unfolding over time (Ohly et al., 2010; Fisher and To, 2012). Finally, ESM and diary methods have been extensively used in work and organizational psychology research to study health and stress (Tennen et al., 2000; Jones et al., 2007), emotions at work (Bono et al., 2007; Jones and Youngs, 2012) and psychological contracts (Conway and Briner, 2002; Griep et al., 2016c).

Participants received short surveys via email in the morning (11 a.m.) and in the afternoon (4 p.m.), and were required to complete the surveys within 2 h after having received the email. As all respondents had a personal computer with internet access for the purpose of their job, it provided us with an inexpensive and effective means for sending prompts and requiring reports (Kubiak and Krog, 2011). Using computers over PDA's, tablet computers or cellphones moreover allows for a larger geographical dispersion of the research sample (Andrews et al., 2011), the use of more detailed anchors, and longer and open-ended questions (Fisher and To, 2012). Only employees who had been actively involved in organizational activities during that day were required to fill out the surveys. All other employees were directed to the end of the survey. We coded responses as missing data when they failed to (timely) complete the survey. We prepared all surveys in Dutch and had three colleagues back-translate the items to English. Inconsistencies between the translation and back-translation were discussed and resolved.

#### Sample

In total, 57 employees were contacted, out of which 51 completed the surveys (89.5% response rate). Note that the unit of analysis is “observations” rather than “respondents,” resulting in 730 valid responses out of a potential maximum of 1,020 observations (51 respondents × 10 days × 2 moments). Respondents were, on average, 38 years old (*SD* = 10.78), 53.19% was female, 78.73% had a higher educational degree, and their tenure was 15.15 years (*SD* = 9.94).

#### Measures

##### General survey measures

We used a general online survey to collect demographic information on respondents' age (in years), gender (female or male), educational background (highest level of formal education), and company tenure (in years).

##### Experience sampling surveys

To clarify that respondents were required to report about the morning or the afternoon, we adapted all items to include “this morning” or “this afternoon” and used past tense (e.g., “this morning I *felt* relaxed”). Where available, we used shortened scales to ensure a reasonable survey length and to avoid jeopardizing respondent compliance. Moreover, we counterbalanced scales to exclude potential order effects (Fisher and To, 2012).

##### Pc breach perceptions

PC breach perceptions were measured using the 3-item measure by Tekleab and Taylor (2003). An example item is “My organization failed to meet its obligations to me this morning.” Respondents indicated the extent to which they agreed with each statement on a 5-point Likert scale ranging from 1 (*totally disagree*) to 5 (*totally agree*). We estimated the between- and within-person reliability based on a multilevel confirmatory factor analysis (Geldhof et al., 2014). The between-person (ω = 0.87) and the within-person (ω = 0.77) reliability estimates were good.

##### Negative emotions

Negative emotions were measured using a 4-item scale by Beal et al. (2013). We asked respondents to indicate the extent to which they had experienced the following emotional states: “anger,” “frustration,” “guilt,” and “unhappiness” on a 5-point Likert scale, ranging from 1 (*not at all)* to 5 (*extremely*). The between-person (ω = 0.94) and the within-person (ω = 0.72) reliability estimates were good.

##### Stress

Stress was measured using the 6-item Stress-in-General Scale (Fuller et al., 2003). Respondents indicated the extent to which their experience of stress reflected the presented adjectives on a 9-point Likert scale ranging from 1 (*not at all)* to 9 (*definitely)*. Four of the items were worded positively (“relaxed,” “calm,” “comfortable,” and “smooth-running”), while two items were worded negatively (“pushed,” and “more stressful than I'd like”). The between-person (ω = 0.92) and the within-person (ω = 0.91) reliability estimates were good.

##### Fatigue

Fatigue was measured with a 2-item measure by Beal et al. (2013). We asked respondents to indicate the extent to which they felt “exhausted” and “energetic” on a 5-point Likert scale, ranging from 1 (*not at all)* to 5 (*extremely*). The between-person (ω = 0.92) and the within-person (ω = 0.84) reliability estimates were good.

#### Analysis

Because respondents provided ratings twice per day for 10 consecutive workdays our data theoretically has a nested structure with four levels: measurements (level 1) nested within days (level 2) nested within weeks (level 3) nested within individuals (level 4). We calculated Intraclass Correlation Coefficients (ICCs) to assess the amount of variance in the variables at these four levels. These ICC values indicated that the majority of variance in perceptions of PC breach (level 1: 27.21%, level 2: 0%, level 3: 0%, level 4: 72.79% of the variance), negative emotions (level 1: 42.41%, level 2: 0.20%, level 3: 0%, level 4: 57.39% of the variance), stress (level 1: 60.59%, level 2: 0%, level 3: 0.28%, level 4: 39.13% of the variance), and fatigue (level 1: 67.67%, level 2: 0.22%, level 3: 0.09%, level 4: 32.02% of the variance) could be attributed to momentary (level 1) and individual (level 4) differences. Because the amount of variance attributed to daily (level 2) and weekly (level 3) differences was lower than 5% and thus negligible (Marcoulides and Schumacker, 2009), we decided to person-mean center all items to remove between-person variance because our hypotheses pertained only to the within-person level.

In practice, this means that we analyzed the data using a 2-level cross-lagged moderated mediation model in Mplus version 7.1 (Muthén and Muthén, 2013). The use of a cross-lagged model means that we computed time-lagged variables. In particular, we used the morning measures (T1) to predict the afternoon measures (T2). We controlled for autoregressive effects [e.g., stress in the afternoon (T2) was regressed on stress in the morning (T1)], consequently controlling for stability in the dependent variables. We followed the recommendations of Edwards and Lambert (2007) and simultaneously tested moderation and mediation effects. The moderation effects were tested by including an interaction between (1) PC breach (Time T) and fatigue (Time T), and (2) negative emotions (Time T+1) and fatigue (Time T). To interpret these multilevel moderation relationships, we person-centered the variables included in the interaction and used the regions of significance approach or the Johnson–Neyman technique (Preacher et al., 2006) instead of the traditional simple slopes method^1^. Despite its broad usefulness, the simple slopes method has an important limitation that largely hampers our ability to interpret the full extent of the interaction: the choices of the conditional values are ultimately arbitrary (i.e., −1*SD*, mean, +1*SD*). The Johnson–Neyman technique identifies the full range of the moderator for which the interaction is significant (i.e., all values where the 95% confidence bands do not include zero). While the upper dashed line in such plots indicates the 2.5% upper region boundaries of significance, the lower dashed line indicates the 2.5% lower region boundaries of significance. The solid line in between the confidence bands represents the size and the direction of the relationship between the independent and the dependent variable for different values of the moderator. The mediation effect was tested by means of the product-of-coefficients approach and its significance was scrutinized by means of the integration algorithm and 95% Confidence Intervals (95%CI).

### Results

#### Multilevel confirmatory factor analysis (MCFA)

We started by performing a MCFA to assess the construct validity of our measures. The theoretical MCFA model fitted the data well, with each item loading significantly and in the expected direction onto its respective latent factor. Alternative model A [i.e., negative emotions and stress load onto one latent factor; PC breach and fatigue each load onto a separate latent factor; $\Delta \chi_{\left(3\right)}^{2}$ = 195.59, *p* < 0.001], alternative model B [i.e., PC breach and negative emotions load onto one latent factor; stress and fatigue each load onto a separate latent factor; $\Delta \chi_{\left(21\right)}^{2}$ = 1927.30, *p* < 0.001], and alternative model C [i.e., PC breach, negative emotions, stress, and fatigue load onto one single latent factor; $\Delta \chi_{\left(6\right)}^{2}$ = 571.69, *p* < 0.001] fit the data significantly worse (see Table 1). Therefore, we are confident that each set of items can be used to assess its respective latent variable and that a 4-factor model can be used for further steps of data analysis.

**Table 1:** Results from multilevel confirmatory factor analyses (Study 1).

**Model**	***χ^*2*^ (df)***	**RMSEA**	**CFI**	**TLI**	**SRMR (within)**
Theoretical model	310.06 (84)	0.06	0.89	0.88	0.06
Alternative model A	505.65 (87)	0.08	0.80	0.76	0.09
Alternative model B	2237.36 (105)	0.08	0.81	0.77	0.08
Alternative model C	881.96 (90)	0.11	0.63	0.57	0.12

*Theoretical model: PC breach, negative emotions, stress, and fatigue each load onto a separate latent factor*.

#### Descriptive results

Table 2 provides an overview of the means, standard deviations, zero-order (between-person) and person-centered (within-person) correlations for all variables under study.

**Table 2:** Means, standard deviations, zero-order and person-centered correlations (Study 1).

	***M***	***SD***	**1**	**2**	**3**	**4**
1. Psychological contract breach	1.65	3.69	–	0.35^***^	0.30^***^	0.55^***^
2. Negative emotions	1.62	0.81	0.30^***^	–	0.62^***^	0.69^***^
3. Stress	4.56	1.74	0.23^***^	0.52^***^	–	0.53^***^
4. Fatigue	2.60	1.00	0.36^***^	0.47^***^	0.48^***^	–

*** *p < 0.001. Zero-order (between-person; N = 51) correlations are presented above the diagonal, whereas person-centered (within-person; N = 417) correlations are presented below the diagonal*.

#### Hypothesis testing

Prior to presenting the results, we estimated a 2-level full moderated mediation model and a 2-level partial moderated mediation model to determine which model offered the best fit to the data. When comparing the Bayesian Information Criterion (BIC)—representing the balance between the number of parameters (i.e., model complexity) and the fit of the model to the data—the BIC value identified the 2-level full moderated mediation model as the one that fits the data best (BIC = 8354.71) compared to the 2-level partial moderated mediation model (BIC = 8355.22).

**Figure 2:**
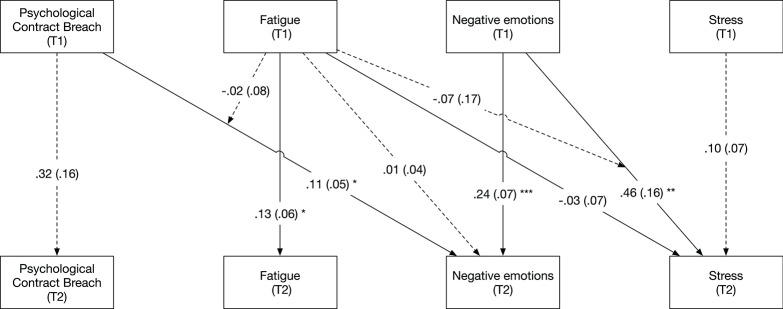
Estimated paths in 2-level cross-lagged moderated mediation model (Study 1). Standard errors between parentheses. Normal lines indicate significant relationships, whereas dotted lines indicate non-significant relationships. Concurrent correlations between T1 and T2 variables were estimated, but are not included in the figure for reasons of parsimony. ^*^p < 0.05, ^**^p < 0.01, ^***^p < 0.001.

Figure 2 depicts the results of the 2-level moderated mediation model. Our results indicated that perceptions of PC breach during the morning were positively related to the experience of negative emotions during the afternoon (β = 0.11, CI_95%_ = [0.01; 0.21]). The experience of negative emotions during the morning was positively related to the experience of stress in the afternoon (β = 0.46, CI_95%_ = [0.15; 0.76]). In addition, our results indicated that being fatigued in the morning was not significantly related to the experience of negative emotions (β = 0.01, CI_95%_ = [−0.07; 0.09]) and stress (β = −0.03, CI_95%_ = [−0.17; 0.12]) in the afternoon. Likewise, we found that the relationship between perceptions of PC breach in the morning and the experience of negative emotions in the afternoon was not moderated by fatigue in the morning (β = −0.02, CI_95%_ = [−0.17; 0.13]), while the relationship between negative emotions in the morning and stress in the afternoon was also not moderated by fatigue (β = −0.07, CI_95%_ = [−0.40; 0.25]). Given that these interaction effects were not significant, we could not find support for Hypotheses 2 and 3.

Finally, we proceeded to examine the cross-lagged indirect effect of psychological contract breach perceptions on stress, mediated by negative emotions. This indirect effect was positive and significant (est. = 0.05, *p* < 0.05), meaning that perceptions of breach triggered an increase in stress, because of increased negative emotions. We could hence support Hypothesis 1.

### Discussion

Results from Study 1 indicated that negative emotions mediate the relationship between PC breach and stress, confirming our first hypothesis. This means that stress, akin to several attitudinal and behavioral outcomes (for a meta-analysis see Zhao et al., 2007), is more likely to increase following negative emotions resulting from PC breach. This finding also indicates that an employee may indeed perceive a PC breach as a (potential) loss of valued resources that will threaten current or future (work) achievements, and hence perceives this to be stressful (Restubog et al., 2013). However, we did not find support for the interaction effect of employees' initial fatigue levels with both PC breach and negative emotions. It is possible that fatigue does not moderate the emotional response to PC breach, but instead reduces the likelihood of perceiving breaches. For example, fatigued employees may be less vigilant when monitoring for potential breaches (Morrison and Robinson, 1997). Alternatively, our measure of PC breach may not have been sensitive enough, as it only focused on breach as receiving less than obligated.

Despite the strengths associated with this first study, we performed a second study to address some limitations. First, our measure of PC breach did not allow us to assess the full continuum of PC breach, including both under- and over-fulfillment. Therefore, Study 2 used a direct comparison measure of PC breach that makes it not only possible to assess the full continuum of PC breach but also to evaluate the intensity of the breach (i.e., the extent to which an employee received each of the PC inducements relative to the extent to which that employee perceived these PC inducements as being promised by the employer). Second, the measure of negative emotions was global in nature. Hence, Study 2 deployed a measure of job related negative affect to better capture the relevant mixture of negative emotions such as disgust, anger, and furious (Morrison and Robinson, 1997). Finally, given that Study 1 was conducted among Belgian employees, Study 2 relied on a sample of US employees from diverse organizations to increase the generalizability of our results.

## Study 2

### Method

#### Procedure

As in Study 1, we used an ESM in which employees from various US organizations (private and public sector) reported on their momentary experiences at two moments per day for 5 consecutive working days. We sent the first survey at a random time between 2.00 and 2.30 p.m. and the second survey at a random time between 6.00 and 6.30 p.m. Respondents were required to respond to the first prompt before 4.00 p.m. (i.e., to ensure that both prompts were at least 2 h apart) and to respond to the second prompt before 8.00 p.m. Only employees who had been actively involved in organizational activities during that day were required to fill out the surveys. All other employees were directed to the end of the survey. We coded responses as missing data when they failed to (timely) complete the survey.

#### Sample

We invited a random group of 78 participants to take part in this study, of whom 53 respondents completed the survey (response rate = 67.94%). The effective sample size included 374 observations (53 respondents x average of 7.06 responses per individual). Respondents were, on average, 48.21 years old (*SD* = 9.81), 43.86% were female, 78.10% obtained a higher educational degree, 34.50% had managerial responsibilities, 81.80% had a permanent full-time contract, and the average tenure was 11.70 years (*SD* = 8.87). Respondents were from diverse industry sectors (top three presented here): education (18.20%), service (14.50%), and manufacturing (10.90%).

#### Measures

##### General survey measures

As in Study 1, we used a general survey to collect demographic information.

##### Experience sampling measures

As in Study 1, we adapted all items to include “this morning” or “this afternoon” and used past tense (e.g., “this morning I *felt* relaxed”), used shortened scale to ensure a reasonable survey length and to avoid jeopardizing respondent compliance, and counterbalanced scales to exclude potential order effects (Fisher and To, 2012).

##### PC breach perceptions

PC breach perceptions were measured using a direct comparison approach (see **Appendix 1**; Turnley and Feldman, 1999; Montes and Irving, 2008). We presented respondents with 13 commonly studied PC items (PSYCONES, 2005) and asked them to indicate to what extent they actually received each of these inducements during the past morning or afternoon compared to the extent to which each of these inducements was previously promised to them, on a 5-point Likert scale ranging from 1 (*received much less than promised*) to 5 (*received much more than promised*). This variable was recoded so that high scores reflect PC under-fulfillment. The between-person (ω = 0.80) and the within-person (ω = 0.93) reliability estimates were good.

##### Negative emotions

Negative emotions were operationalized using the ten negative emotion items of the job-related affective well-being scale (JAWS; Van Katwyk et al., 2000). Respondents rated the extent to which they felt each of the negative emotions (e.g., anger, frustration) during the past morning or afternoon on a 7-point Likert scale, ranging from 1 (*minimally to not at all*) to 7 (*to a very great extent*). The between-person (ω = 0.73) and the within-person (ω = 0.93) reliability estimates were good.

##### Stress and fatigue

Stress and fatigue were measured using identical measures as in Study 1. The between-person and the within-person reliability estimates for stress (ω = 0.94 and ω = 0.90, respectively) and fatigue (ω = 0.80 and ω = 0.52, respectively) were good.

#### Analysis

Because participants provided ratings twice a day for 5 consecutive working days, our data has a nested structure with three levels: measurements (level 1) nested within days (level 2), nested within individuals (level 3). To account for this nested structure, we again calculated ICCs to assess the amount of variance in the variables at the three levels. These ICC values indicated that most of the variance of PC breach (level 1: 0.27%, level2: 0%, level 3: 0.73% of the variance), negative emotions (level 1: 19%, level 2: 0.1%, level 3: 81%), stress (level 1: 39%, level 2: 1%, level 3: 60% of the variance), and fatigue (level 1: 35%, level2: 0.1%, level 3: 65% of the variance) was situated at the between-person level (level 3) or at the momentary level (level 1). Because the amount of variance attributed to daily (level 2) differences was lower than 5%, and thus negligible (Marcoulides and Schumacker, 2009), we decided to person-mean center all items to remove between-person variance because our hypotheses pertain only to the within-person level. The same procedure was followed as in Study 1 when analyzing our data using a 2-level moderated mediation model in Mplus version 7.1 (Muthén and Muthén, 2013). The only exception to this procedure pertains to the inclusion of a curvilinear PC breach effect (i.e., interaction between PC breach and itself at the same point in time), to test the full spectrum of PC breach, ranging from under- to over-fulfillment.

### Results

#### Multilevel confirmatory factor analysis (MCFA)

We started by performing a MCFA to assess the construct validity of our measures. The theoretical MCFA model fitted the data well, with each item loading significantly and in the expected direction onto its respective latent factor. Alternative model A [i.e., negative emotions and stress load onto one latent factor; PC breach and fatigue each load onto a separate latent factor; $\Delta \chi_{\left(3\right)}^{2}$ = 504.34, *p* < 0.001], alternative model B [i.e., PC breach and negative emotions load onto one latent factor; stress and fatigue each load onto a separate latent factor; $\Delta \chi_{\left(3\right)}^{2}$ = 798.08, *p* < 0.001], and alternative model C [i.e., PC breach, negative emotions, stress, and fatigue load onto one single latent factor; $\Delta \chi_{\left(4\right)}^{2}$ = 1206.41, *p* < 0.001] fit the data significantly worse (see Table 3). Therefore, we are confident that each set of items can be used to assess its respective latent variable and that a 4-factor model can be used for further steps of data analysis. However, it should be noted that for the analysis of negative emotions, we did not include one item (i.e., fatigued) of the original 10 items because of its high relatedness to the moderating variable fatigue.

**Table 3:** Results from multilevel confirmatory factor analyses (Study 2).

**Model**	***χ^*2*^ (df)***	**RMSEA**	**CFI**	**TLI**	**SRMR (within)**
Theoretical model	1508.28 (95)	0.08	0.93	0.91	0.13
Alternative model A	2012.62 (92)	0.10	0.81	0.58	0.15
Alternative model B	2306.36 (92)	0.11	0.54	0.50	0.18
Alternative model C	2714.69 (89)	0.12	0.44	0.40	0.18

*Theoretical model: PC breach, negative emotions, stress, and fatigue each load onto a separate latent factor*.

#### Descriptive results

Table 4 provides an overview of the means, standard deviations, zero-order (between-person) and person-centered (within-person) correlations for all variables under study.

**Table 4:** Means, standard deviations, zero-order and person-centered correlations (Study 2).

	***M***	***SD***	**1**	**2**	**7**	**8**
1. Psychological contract breach	3.04	0.45	–	0.01	0.39^**^	0.13
2. Negative emotions	1.51	0.76	0.16^**^	–	0.65^***^	0.44^***^
3. Stress	3.22	1.64	0.41^***^	0.59^***^	–	0.60^***^
4. Fatigue	2.73	95	0.18^***^	0.31^***^	0.48^***^	–

** p < 0.01;

*** *p < 0.001. Zero-order (between-person; N = 53) correlations are presented above the diagonal, whereas person-centered (within-person; N = 374) correlations are presented below the diagonal*.

#### Hypothesis testing

As in Study 1, we started by estimating a 2-level full moderated mediation cross-lagged model and a 2-level partial moderated mediation cross-lagged model to determine which model offered the best fit to the data. Both models contained both a linear and a curvilinear effect of psychological contract breach. The 2-level full moderated mediation fits the data best (BIC = 3398.86) compared to the 2-level partial moderated mediation model (BIC = 3408.48).

**Figure 3:**
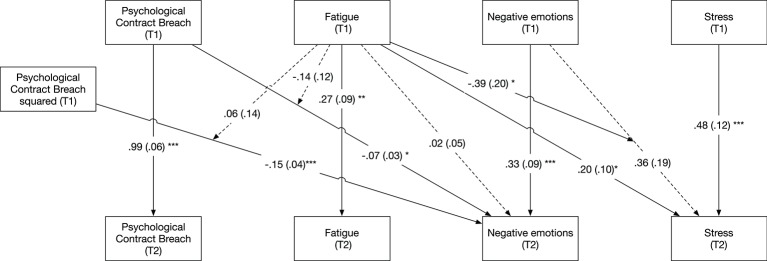
Estimated paths in 2-level cross-lagged moderated mediation model (Study 2). Standard errors between parentheses. Normal lines indicate significant relationships, whereas dotted lines indicate non-significant relationships. Concurrent correlations between T1 and T2 variables were estimated, but are not included in the figure for reasons of parsimony. ^*^p < 0.05, ^**^p < 0.01, ^***^p < 0.001.

**Figure 4:**
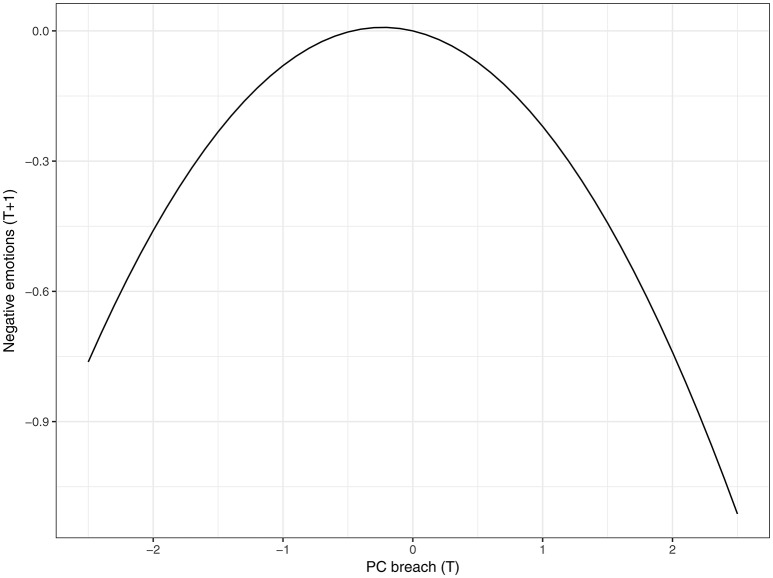
Curvilinear effect of psychological contract breach perceptions during the morning on negative emotions in the afternoon. Negative values for psychological contract breach represent over-fulfillment, zero represents fulfillment, and positive values represent under-fulfillment.

Figure 3 depicts the results of the 2-level cross-lagged full moderated mediation model. Our results indicated that the linear effect of perceptions of PC breach in the morning was negatively related to the experience of negative emotions during the afternoon (β = −0.07, CI_95%_ = [−0.13; −0.001]). Moreover, there was a significant negative curvilinear effect of PC breach perceptions in the morning on negative emotions in the afternoon (β = −0.15, CI_95%_ = [−0.23; −0.07]). To interpret this curvilinear effect, we plotted the relationship between negative emotions in the afternoon and perceptions of PC breach in the morning (see Figure 4). As can be seen in Figure 4, there is a decrease in negative emotions experienced during the afternoon, when employees experience both under- and over-fulfillment in the morning. The experience of negative emotions during the morning was positively related to the experience of stress during the afternoon, albeit only marginally significant (β = 0.36, CI_95%_ = [−0.01; 0.72], CI_90%_ = [0.05; 0.66]). In addition, our results indicated that the experience of fatigue during the morning was not related to the experience of negative emotions (β = 0.02, CI_95%_ = [−0.07; 0.11]), while it was significantly related to stress (β = 0.20, CI_95%_ = [0.003; 0.40]) during the afternoon.

**Figure 5:**
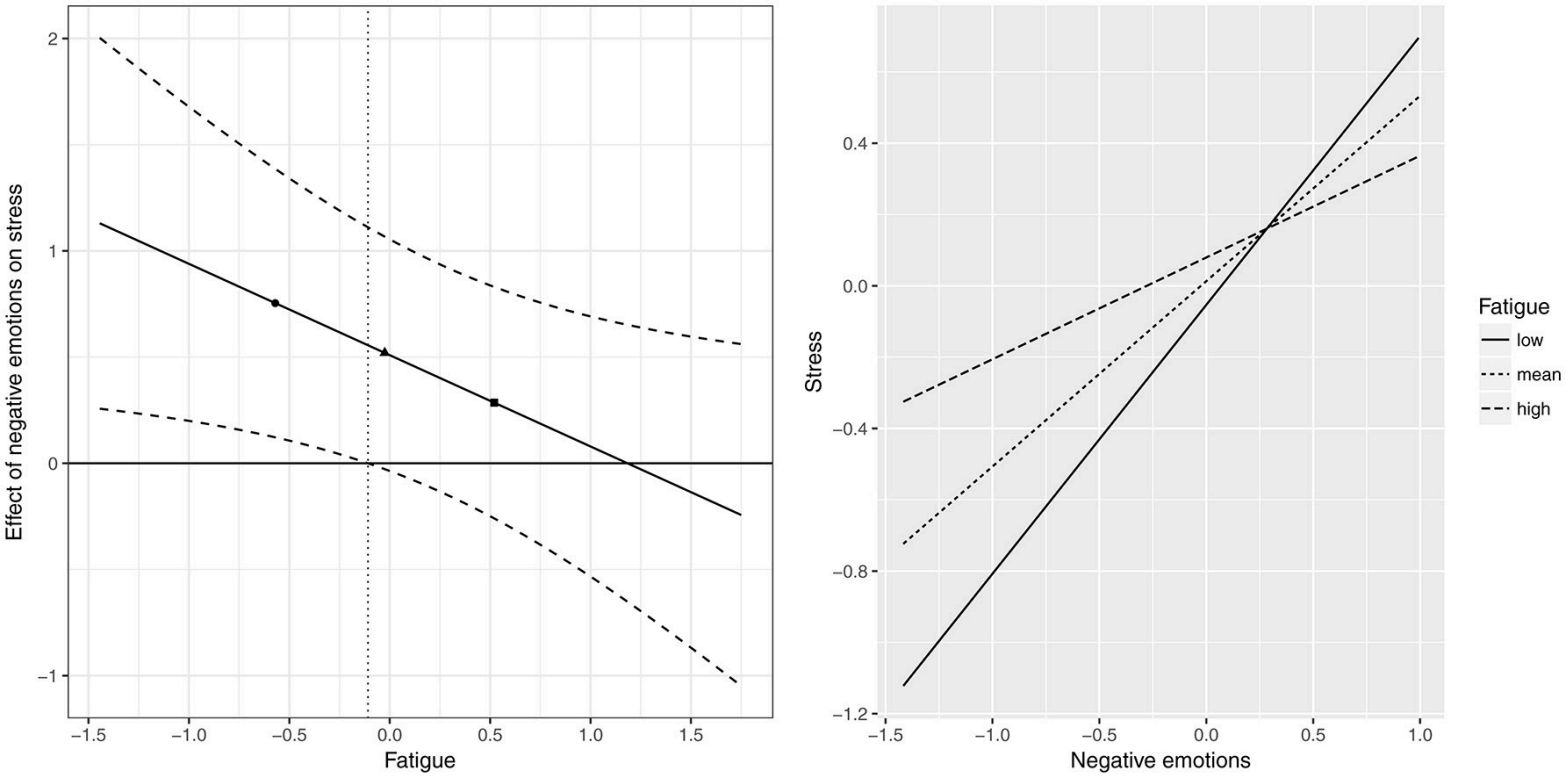
Johnson–Neyman plot (**left** side) and Aiken and West simple slope plot (**right** side) for the 2-level moderating role of fatigue in the relationship between negative emotions and stress. Note that the first symbol (circle) corresponds to low levels of fatigue (−1*SD*), the second symbol (triangle) corresponds to mean levels of fatigue (mean equals zero), and the third symbol (square) corresponds to high levels of fatigue (+1*SD*). In the Johnson-Neyman plot, there is a significant positive relationship between negative emotions and stress for any value of fatigue on the left-hand side of the vertical dotted line.

Next, we found that the relationship between negative emotions in the morning and stress in the afternoon was moderated by the experience of fatigue in the morning (β = −0.39, CI_95%_ = [−0.78; −0.01]), whereas the linear and curvilinear effect of PC breach in the morning on negative emotions in the afternoon was not moderated by fatigue experienced during the morning (β = −0.14, CI_95%_ = [−0.36; 0.09] and β = 0.06, CI_95%_ = [−0.21; 0.34], respectively), thus offering no support for Hypothesis 2. Figure 5 shows the Johnson–Neyman and simple slopes plots for the moderating role of fatigue (time T) on the relationship between negative emotions (time T) and stress (time T+1). As can be seen in this figure, there was a significant positive relationship between negative emotions and stress, when fatigue was low (est. = 0.57, *p* < 0.05). This positive relationship became weaker as fatigue increased, with non-significant effects for average (est. = 0.36, *p* = 0.05) and high (est. = 0.15, *p* = 0.53) values of fatigue. More precisely, there was a significant positive relationship between negative emotions and stress, for person-mean centered values of fatigue lower than −0.01. While the presence of this interaction effect aligns with Hypothesis 3, the direction of the effect is opposite to what we hypothesized.

Finally, we estimated time-lagged conditional indirect linear and curvilinear effect of perceptions of PC breach on the experience of stress, via negative emotions, for low (−1*SD*), average, and high (+1*SD*) values of fatigue. There were no significant linear indirect effects of breach perceptions on stress, via negative emotions, when fatigue was low (est. = −0.04, *p* = 0.11), average (est. = −0.02, *p* = 0.18), or high (est. = −0.01, *p* = 0.55). However, there was a significant curvilinear indirect effect of breach perceptions on stress, via negative emotions, when fatigue was low (est. = −0.08, *p* < 0.05). This curvilinear indirect effect became non-significant when fatigue was average (est. = −0.05, *p* = 0.11) or high (est. = −0.02, *p* = 0.54). These results suggest the presence of an ∩-shaped indirect effect when fatigue is low, meaning that breach perceptions in the morning (both in the form of under- and over-fulfillment) relate to a decrease in stress in the afternoon. In sum, we can partially support Hypothesis 1 as we find evidence for an indirect effect of breach perceptions on stress via negative emotions, but only for low values of fatigue.

#### Sensitivity analysis: testing concurrent effects

**Figure 6:**
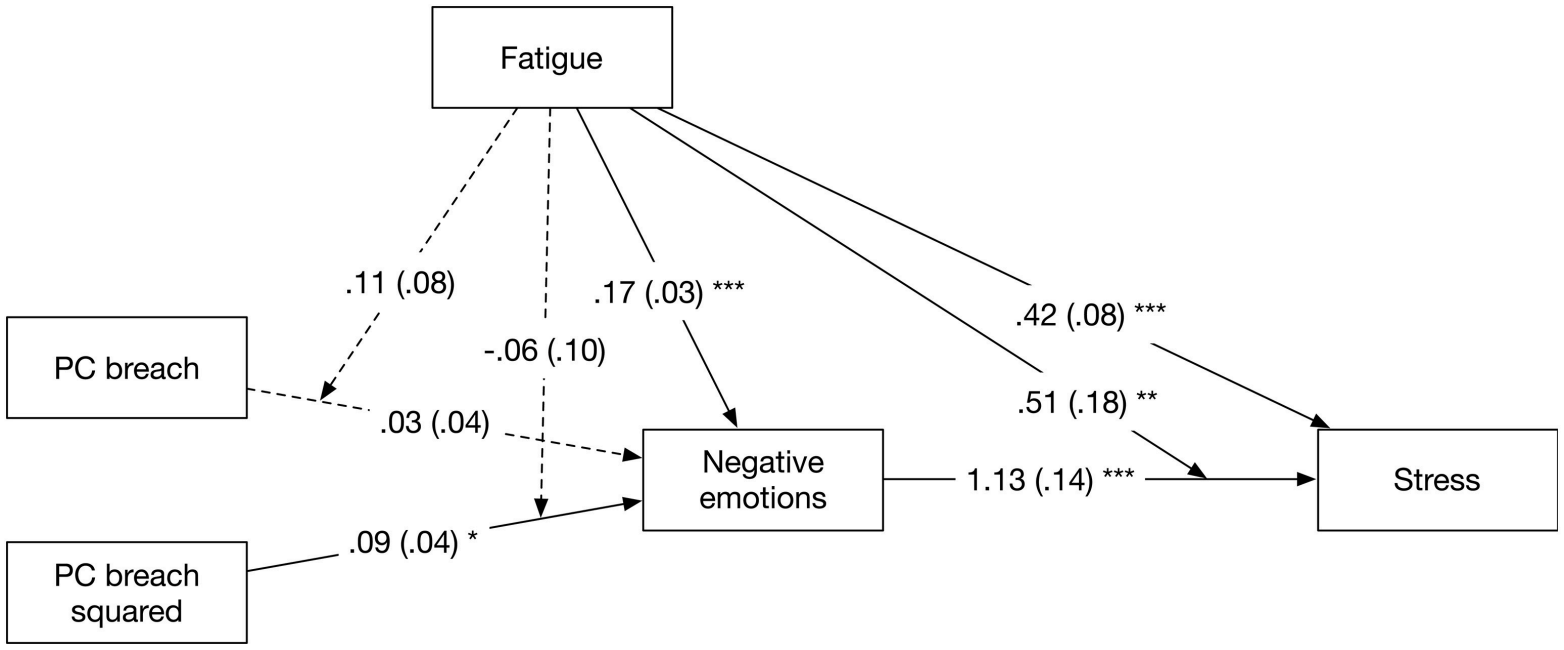
Path estimates from model with concurrent effects in Study 2. Standard errors between parentheses. Normal lines indicate significant relationships, whereas dotted lines indicate non-significant relationships. ^*^p < 0.05, ^**^p < 0.01, ^***^p < 0.001.

The time-lagged effects of breach perceptions on negative emotions, and indirectly on stress, reported above appear counter-intuitive at first glance, because they suggest that under- and over-fulfillment related to decreases in negative emotions and stress. However, one possible explanation for this is that the time-lag that we introduced allowed employees to recover from any immediate negative consequences from PC breach perceptions. We therefore re-estimated the full moderated mediation model, using only concurrent effects (i.e., all variables are measured at the same point in time). Figure 6 shows the path estimates from this model. As can be seen in this figure, the curvilinear effect of PC breach perceptions was significantly and positively related to negative emotions (β = 0.09, CI_95%_ = [0.02; 0.17]). Fatigue was also significantly and positively related to negative emotions (β = 0.17, CI_95%_ = [0.11; 0.24], whereas the linear effect of breach and the interaction between the linear and curvilinear effects of breach and fatigue were not significant. Negative emotions were, in turn, positively related to stress (β = 1.13, CI_95%_ = [0.86; 1.39]). Moreover, fatigue (β = 0.42, CI_95%_ = [0.26; 0.57]) and the interaction between negative emotions and fatigue (β = 0.51, CI_95%_ = [0.16; 0.85]) were significantly related to stress. Examination of the conditional indirect effects revealed that the linear indirect effect of breach perceptions was not significantly related to stress, via negative emotions, for low (est. = 0.03, *p* = 0.36), average (est. = 0.04, *p* = 0.36), or high (est. = 0.05, *p* = 0.36) values of fatigue. The curvilinear effect of breach perceptions was significantly and positively related to stress, via negative emotions, and become stronger as fatigue went from low (est. = 0.08, *p* < 0.05), to average (est. = 0.11, *p* < 0.05), to high (est. = 0.13, *p* < 0.05) values. In summary, we find support for Hypotheses 1 and 3 when examining concurrent relationships.

### Discussion

The inclusion of a direct comparison measure in Study 2 offered a more fine-grained understanding of the mediation effect of negative emotions in the PC breach—stress relationship. Specifically, we found that negative emotions mediate the relationship between stress and both under- and over-fulfillment. However, the direction of this effect is opposite to what we hypothesized. Namely, our findings suggest that experiencing a breach (both under- and over-fulfillment) will lead to less negative emotions and stress. The time-lag employed in this study might have caused this counterintuitive finding. Indeed, employees were allowed to fill in the questionnaire until 8 pm. This time-lag could have as a consequence that employees had enough time to recover from the previous experienced breach. Moreover, given the time of the day, employees probably responded to the questions at home, which may lead to potential effects of work detachment, and in turn less intense negative emotions and stress. Another plausible explanation would be that employees were during the completion of the survey too fatigued to experience negative emotions or stress. This might consequently lead to tainted results. Therefore, we decided to also test the hypothesized moderation and mediation effects at the concurrent level. This sensitivity analysis revealed that negative emotions and stress immediately increase when employees perceive not only under- but also over-fulfillment, confirming our first hypothesis. Moreover, this mediated relationship became stronger when employees were more fatigued.

In view of hypothesis 2, we couldn't demonstrate the moderating effect of fatigue on the positive PC breach–negative emotions relationship. Nonetheless, we did find a direct positive effect of being fatigued on the experience of negative emotions. This finding suggests that simply being fatigued, even in the absence of a PC breach results in the experience of negative emotions.

In contrast to Study 1, we were able to confirm that the relationship between negative emotions and stress is moderated by one's level of fatigue, supporting Hypothesis 3. This finding implies that an employee will experience more stress along with the experience of negative emotions when that employee is additionally experiencing high levels of fatigue. Indeed, an employee who faces an affective work event (i.e., PC breach) that triggers negative emotions will have to engage in emotion-regulation efforts that require the availability of (energetic) resources to effectively deal with this negative work event (Gross, 2002). However, if these resources are already drained by for example being fatigued, that employee may be unable to alter his/her negative emotions and apply adequate coping styles to reduce elicited stress (Hobfoll, 2001).

## General discussion

In the current 2-study paper, we aimed to investigate the dynamics that relate perceptions of PC breach to negative emotions and experiences of stress. We contributed to the literature by adopting a within-person process perspective to understand how perceiving PC breach elicits increases in stress resulting from negative emotions within the same individual, compared to when that individual does not perceive PC breach. Moreover, we simultaneously examined mediating (negative emotions) and moderating (fatigue) mechanisms of the PC breach—stress relationship to provide a more detailed understanding of the interplay between PC breach and stress. In doing so, we aimed to further integrate the PC and stress literatures.

### Theoretical implications

A first main implication of both studies pertains to the finding that PC breach can trigger stress reactions through negative emotions. The mediating role of negative emotions in the positive PC breach—stress relationship aligns with propositions from COR Theory (Hobfoll, 1989); perceptions of PC breach may be interpreted as an actual or perceived loss of valued resources, which in turn may lead employees to experience negative emotions, resulting in elevated stress levels (Restubog et al., 2013). Furthermore, by employing a direct comparison measure of PC breach in Study 2, we can further nuance this finding: both under- and over-fulfillment of PC breach will immediately evoke negative emotions and elicit stress reactions. This finding is in accordance with previous research (Lambert et al., 2003; Vantilborgh et al., 2014) showing that excess inducements may also lead to negative outcomes. For instance, if high levels of inducements interfere with the fulfillment of other needs and desires, they become harmful to the employee (Edwards et al., 1998). For example, high levels of task variety may interfere with an employee's need to develop proficiency on a core set of skills, consequently averting task performance (Lambert et al., 2003). In terms of resources, it can be argued that if over-fulfillment of the PC is associated with a loss or a threat of important resources, it will lead to elevated stress levels resulting from experienced negative emotions (Hobfoll, 2001).

Beyond merely demonstrating a positive association between PC breach and stress via negative emotions, the second study suggested, albeit at the concurrent level, that this mediation relationship grew stronger when an employee was more fatigued and hence thus not possess sufficient energy resources to cope with this negative work event. Not only do these findings align with the aforementioned principles of COR Theory (1989), they also align with the behavioral economics premise that the balance between energy expenditure and recovery in stressful circumstances will determine the intensity of the emotional reaction (Schönpflug and Battmann, 1988). Both COR Theory and the aforementioned behavioral economics premise indicate that the levels of energy an employee has available at the moment (s)he experiences a PC breach will influence the intensity of both the emotional and the stress reaction following this PC breach. If an employee has a low level of energy, negative emotions and stress associated with PC breach are expected to increase because these events further drain the already exhausted resources (Hobfoll, 2001). Cognitive appraisal will then indicate a more severe threat emerging from the smaller odds of successful coping results (Lazarus, 1991). As such, the feedback regarding the size of discrepancy (Kluger and DeNisi, 1996) and the meta-feedback regarding the rate of discrepancy reduction (Carver and Scheier, 1990) will lead to further discomfort. Overall, these theoretical propositions indicate that employees will be more prone to experience stronger negative emotions and higher stress levels when PC breaches are perceived in high fatigue circumstances.

### Limitations and future directions

Notwithstanding the theoretical and methodological contributions of this paper, some limitations need to be taken into account. First, we could not replicate the time-lagged mediation effect found in our first study, leading us to tests our hypotheses at the concurrent level. However, the time-lags we employed in our second study may explain this finding, as they may have allowed employees to detach from work and recover from breaches. Therefore, we advise future research to not only use shorter intervals between two consequent prompts to capture recovery processes, but also to use time-lags that fall within employees' working hours in order to avoid possible work detachment effects. Moreover, we advocate for the use of experimental designs to establish the causality of the PC breach–negative emotions–stress relationship.

Second, the self-reported nature of our measures might raise concerns about common method bias and social desirability (Podsakoff et al., 2012). However, we tried to minimize risks pertaining to social desirability by guaranteeing confidential and discrete participation. Moreover, several scholars argue that self-reports are less problematic when the research focusses on within-person, instead of between-person, differences (see Beal and Weiss, 2003). We aimed to reduce concerns about common method bias by using time-lags, and by presenting all scales in a random order. Finally, Siemsen et al. (2010) argued that common method bias cannot explain or distort interactions effects. Hence, the presence of significant interactions in both studies helps to strengthen our argument that the observed relations are a function of the studied constructs and relationships rather than methodological artifacts.

Third, the intensity of stress and emotional reactions associated with the perception of PC breach are likely influenced by individual and organizational factors such as stress resilience, coping strategies, and organizational social accounts. We therefore advise future research to either control for these factors or explicitly model and investigate their influence.

Finally, although the rather small sample size at the individual level might raise some concerns about the power in our analyses, it is worth mentioning that the unit of analysis is “observations” rather than “respondents,” resulting in more than sufficient sample sizes for the type of analysis used in both studies (for a detailed explanation see Hox, 2010). Moreover, several simulation studies (e.g., Browne and Draper, 2000) underlined the importance of the absolute number of Level 2 units (i.e., respondents) in favor of the ratio of Level 2 to Level 1 (i.e., daily entries) units when guaranteeing sufficient power and accuracy of fixed estimates. When Level 2 units exceed thirty, the statistical model provides an accurate estimate of standard errors and fixed effects (Maas and Hox, 2005). A *post-hoc* Monte Carlo power analysis revealed that we had sufficient power when assessing fixed effects (study 1: average power = 0.71; study2: average power = 0.83), although the power to detect within-person interaction effects was low (study 1: average power = 0.07, study 2: average power = 0.06) (Muthén and Muthén, 2002). However, interaction effects are known to typically have low power (Mathieu et al., 2012).

Considering the generalizability of our findings, it is important to note that we focused on within-person effects rather than on between-person differences, meaning that individual differences can only influence within-person effects as cross-level moderators. Further, we examined the extent to which our findings can be generalized by testing our hypotheses in two samples from different countries (Belgium and the United States). Research suggests that cultural differences may shape employees' reactions to psychological contract breach (Thomas et al., 2003). For example, Belgium is known to score higher on uncertainty avoidance and power distance than the United States (Hofstede, 2001). The relative tolerance for uncertainty may mean that US employees react less strongly to uncertain situations such as a psychological contract breach, whereas the higher power distance may lead to Belgian employees being less likely to perceive psychological contract breaches. The differences found in our two studies may be, partially, due to cultural differences, and we believe that the role of culture in psychological contracts forms an important avenue for future research.

### Practical implications

First and foremost, our results indicate the need for organizations to avoid perceptions of PC breach from arising if they want to improve the well-being of their employees. More specifically, it appears that PC breach triggers elevated stress levels as a result of experienced negative emotions. These emotional and stress reactions are especially strong when employees are fatigued. Organizations should therefore be aware of and assess available resources, thereby being able to train employees how to use these resources effectively. In this regard, it is noteworthy that over-fulfillment of the PC may also lead to stress resulting from negative emotions. Hence, well-intended supervisors who aim to reward their employees by, for example, exceeding obligation of training opportunities may unknowingly create overload leading to elevated stress due to task conflicts and the uncertainty about what the supervisor desires in return from the employees. For instance, employees may be concerned with their lack of time to attend all the training programs or they may be preoccupied by the feasibility of their current job tasks. Therefore, organizations that wish to reward their employees by over-fulfilling obligations should make sure that employees are rested and have sufficient psychological resources to correctly deal with this over-fulfillment.

Furthermore, organizational interventions and social accounts, such as apologies or compensation, may also help to reduce the experience of negative emotions after experiencing PC breach (Lester et al., 2007). Organizations should moreover intervene to repair PC breach because previous studies have shown that perceptions of PC breach can be self-sustaining, meaning that current perceptions of PC breach increase the likelihood to perceive new PC breaches (Griep et al., 2016a,b). This implies that organizations who do not respond to employee perceptions of PC breach may have a workforce that is increasingly perceiving PC breach, ultimately resulting in a chronically stressed workforce.

Finally, previous research demonstrated that stress management is more effective when the intervention focuses both on the individual and the organization (Kompier et al., 2000; McVicar, 2003). In line with this, we suggest that employees can also be trained to be aware of and to cope efficiently with the negative PC breach—stress spiral. As such, learning to apply adequate cognitive reappraisal strategies that allow effective down-regulation of negative emotions emerging from PC breach may serve as an important buffer against high stress (Gross and Muñoz, 1995). For example, employees may be trained to focus on more positive aspects of their PC and/or use remaining job resources in order to reduce the impact of PC breach on emotional and stress reactions (Bakker et al., 2003). Last but not least, based on present research, we suggest that employees should also be trained to become more aware of their own fatigue levels through self-monitoring techniques to prevent further resources depletion and negative loss spirals.

## Author contributions

SA, YG, TV: conceptualization, data curation, formal analysis, investigation, methodology, project administration, resources, software, supervision, validation, visualization, writing ± original draft, and writing ± review and editing.

### Conflict of interest statement

The authors declare that the research was conducted in the absence of any commercial or financial relationships that could be construed as a potential conflict of interest.

## Footnotes

^1^To facilitate interpretation for the reader unfamiliar with the Johnson-Neyman plots, we included simple slopes plots alongside the Johnson-Neyman plots.

## Appendix 1

**Table A1:** Psychological contract items Study 2.

	**Received much less than the obligation (1)**	**Received less than the obligation (2)**	**Received about the same as the obligation (3)**	**Received more than the obligation (4)**	**Received much more than the obligation (5)**
1. Providing career counseling and mentoring					
2. Providing well-defined work roles and tasks					
3. Providing a reasonable workload					
4. Allowing to take part in decision making					
5. Providing sufficient resources to adequately perform the job					
6. Providing desired levels of responsibilities					
7. Approachable managers					
8. Providing an honest and respectful working atmosphere					
9. Managers who show concern for my well-being					
10. Allowing flexible working hours					
11. Providing recognition for my performance					
12. Providing a safe working environment					
13. For this item indicate “Received about the same as obligation”					

*In this section, we are interested in your opinion on the extent to which your organization has fulfilled its obligations to you this morning/afternoon. Think about your experience at work since the beginning of the morning/afternoon. Compare the extent to which you actually received these items with the extent to which you believe that the organization is obliged to give you these matters*.
